# Quantitative abundance and distribution data from the first systematic survey of alien invasive plants in the Xiaoxing'an Mountains, China (2022–2023)

**DOI:** 10.3897/BDJ.14.e178047

**Published:** 2026-01-05

**Authors:** Yutong Zhang, Zhijian He, Xueyun Dong, Liqiang Mu, Hongfeng Wang

**Affiliations:** 1 Northeast Forestry University, Harbin, China Northeast Forestry University Harbin China; 2 Harbin University, Harbin, China Harbin University Harbin China; 3 Northeast Forestry University, Haerbin, China Northeast Forestry University Haerbin China

**Keywords:** north-eastern China, occurrence records, biodiversity, dataset, non-native species

## Abstract

**Background:**

Biological invasions pose a major threat to the ecological stability of the Xiaoxing'an Mountains region, a significant temperate forest ecosystem in north-eastern China. Although effective management and risk assessment are urgently needed, substantial data gaps remain regarding the precise spatial distribution and quantitative abundance of invasive plant species in this area. This dataset aims to address this gap by systematically documenting species occurrences and abundance metrics.

**New information:**

This dataset documents the results of the first systematic survey of invasive plants conducted in the Xiaoxing'an Mountains region of Heilongjiang Province, China, spanning 2022 to 2023. Constructed strictly according to the Darwin Core (DwC) standard, the sampling event dataset comprises 4408 unique sampling events and 4773 invasive plant occurrence records (covering 37 species). The most frequently recorded species were *Erigeron canadensis* (1723 records), followed by *Trifolium repens* (1157 records). Each record is provided with precise geographic coordinates and complete taxonomic identification and the majority of species records include percent coverage information, establishing a solid foundation for regional ecological risk assessment, species distribution modelling and long-term conservation management planning.

## Introduction

Biological invasions are now occurring extensively on a global scale, representing a significant component of global change and exerting profound impacts on the Earth. Invasive plants are not only a leading cause of ecological biodiversity loss, but also pose a significant threat to global ecosystems and economic development.

Regarding biodiversity, invasive species often displace native species from their ecological niches, leading to population declines and even extinctions (Enserink M. 1999, Gurevitch and Padilla 2004, O'Dowd et al. 2010, Yurkonis and Meiners 2010). For instance, *Alternanthera philoxeroides* (Mart.) Griseb. can lead to community homogenisation (Li and Xie 2002), while *Spartina alterniflora* Loisel. competitively displaces native species (Chen et al. 2004). Biological invasion has become the second largest factor contributing to biodiversity loss globally (Wilcove et al. 1998, Reichard and White 2003).

At the ecosystem level, plant invasions can alter interspecific interactions (Vázquez and Simberloff 2003), thereby affecting the structure and ecological functions of populations, communities and entire ecosystems (Elton 1977, Vitousek et al. 1996, Wilcove et al. 1998, Peng and Xiang 1999, Gurevitch and Padilla 2004), posing a significant threat to overall ecosystem stability. Economically, invasive organisms reduce crop yields and increase management costs, leading to severe financial impacts. Furthermore, once an alien species successfully establishes itself, its eradication becomes exceedingly difficult (Zavaleta et al. 2001.) Statistics indicate that the United States incurs annual economic losses of approximately $137 billion due to biological invasions (Pimentel et al. 2000). In China, invasive plants have also imposed severe threats to the economy, ecology, biodiversity, social environment and human livelihood safety. According to comprehensive statistics, alien species cause direct economic losses to agriculture and forestry in China amounting to $7 billion annually (Li and Xie 2002).

The Xiaoxing'an Mountains, a branch of the Changbai Mountain Range, are located in Northeast China and fall within the northern part of the "Temperate Mixed Coniferous and Broadleaf Forest Zone" under China's vegetation classification system. This region features exceptionally high fractional vegetation cover and diverse ecosystem types. Notably, the Xiaoxing'an Mountains preserve China's only remaining pristine Korean pine mixed broadleaf-conifer forest (Zhou 1997), holding immense ecological and scientific research value. However, research on invasive plants in Heilongjiang Province is notably underdeveloped and no systematic investigation of invasive plants has ever been conducted in the Xiaoxing'an Mountains region. Modern genetic studies suggest that species can adapt to new environments in as few as 20 generations or even less (Prentis et al. 2008), indicating that invasive plants could rapidly establish within ecosystems and cause significant damage to native ecology. Therefore, conducting a comprehensive inventory of invasive plants in the Xiaoxing'an Mountains represents an urgent and critical task.

## General description

### Purpose

This dataset is designed to address the current lack of data on the spatial distribution and quantitative abundance of invasive plants in the Xiaoxing'an Mountains region. It provides precise, standardised and quantifiable inventory data to support future research on regional ecological risk assessment, species distribution modelling and the development of effective, evidence-based management strategies for invasive alien species in temperate forest ecosystems.

### Additional information

The current release represents the initial version of the dataset. The standardisation process strictly follows the Darwin Core Archive (DwC-A) standard and the dataset (Zhang 2025) is published via the Integrated Publishing Toolkit (IPT). All records have undergone rigorous quality control checks, including taxonomic verification and validation of geographic coordinate accuracy. Future updates are planned to incorporate additional field survey seasons and expanded taxonomic coverage, ensuring the long-term utility of this dataset as a resource for regional ecological research.

## Project description

### Title

Invasive Plant Occurrence Data from the Xiaoxing'an Mountains, China

### Study area description

The Xiaoxing'an Mountains are located in the northeast of Heilongjiang Province, China, spanning from 125°54′00″E to 130°56′00″E and 46°10′00″N to 51°2′00″N. The range is bordered to the north by the Amur River (Heilongjiang), which separates it from Russia, with a border line stretching 249.5 km.

The region experiences a humid continental monsoon climate within the northern temperate zone, influenced by both maritime warm-moist air currents and the Siberian cold air mass. The climate is characterised by four distinct seasons. The accumulated temperature ≥ 10°C ranges between 1800°C and 2300°C and the dryness index falls between 0.5 and 0.9, classifying it as a temperate humid forest climate. The mean annual temperature hovers around 0°C, with the highest temperatures occurring from June to August and the lowest from December to February, resulting in a substantial annual temperature range. The average annual precipitation is several hundred millimetres, with summer receiving the most rainfall and winter experiencing several months of snow cover (Li Peilin 2023).

Due to the cold climate, sporadic permafrost patches are distributed across the area, with depths ranging from several metres to tens of metres. Although the growing season is limited to 4–5 months, the combination of ample precipitation and sufficient thermal energy during this period creates highly favourable conditions for plant growth and development, contributing to the remarkable diversity of vegetation types in the study area.

The geological structure of the Xiaoxing'an Mountains is complex, situated within the Yichun-Yanshou geosynclinal fold belt of the Khingan-Mongolian fold region. Roughly bounded by the Jiayin-Tieli line, significant geomorphological differences are evident between the north-western and south-eastern parts. The mountain range generally trends from northwest to southeast, with higher elevations in the southeast and lower in the northwest, demonstrating pronounced geomorphic layering. The mountains in the south-eastern part mostly reach elevations between 800 and 1000 metres, with some peaks exceeding 1000 metres and the highest summit reaching 1429 metres (Zhou 1994).

### Design description

The systematic investigation spanned a two-year period from 2022 to 2023, with observations conducted annually during the plant growing season (June to September). The dataset is based on 4408 unique sampling events, yielding a total of 4773 occurrence records for 37 invasive plant species. Every entry in the dataset is accompanied by detailed taxonomic information (including species, genus, family, order, class, phylum and kingdom), as well as comprehensive sampling event details (including date, location and geographic coordinates), with the majority of observations recording the species' coverage.

### Funding

National Key Research and Development Program of China, 2024YFF1307603, "Research and National Practice on the Biodiversity Rapid Assessment System for the "Kunming-Montreal Global Biodiversity Framework".

## Sampling methods

### Sampling description

To systematically document the distribution and abundance of invasive plant species in the diverse ecosystems of the Xiaoxing'an Mountains, we employed two complementary, spatially explicit sampling methods: systematic grid-based surveys and roadside transect surveys. This dual-method design was intended to achieve broad geographic coverage, while intensively sampling high-risk invasion corridors, such as roadsides and disturbed habitats.

### Step description

1. Systematic Grid Survey：This method aimed to obtain spatially balanced and ecologically representative samples independent of the road network.

Grid Design and Site Selection: The study area was evenly divided into a survey grid of 20 km × 20 km cells. Within each cell, one primary survey point was selected. Site selection was not random, but strategically targeted towards areas presumed to have higher invasion susceptibility, such as locations with complex vegetation structure, distinct habitat edges or a history of anthropogenic disturbance.

Fixed-Area Reconnaissance: A 300 m × 300 m square area was established, centred on the geographic coordinates of the sampling point. This predefined area was thoroughly and systematically searched to locate all patches of invasive plants.

Targeted Plot Establishment and Quantitative Recording: When a discrete patch or notable concentration of invasive plants was identified during reconnaissance, a sampling plot was immediately established at that precise location. Plot size was determined by the life form of the species, as follows. Herbaceous plants: A 1 m × 1 m quadrat was used. Shrub plants: A 5 m × 5 m quadrat was used. Within each plot, all invasive plant species were identified to the species level and their total percentage cover was estimated. These records constitute the core quantitative presence-abundance data in our dataset. (Note: As the invasive flora in this region is overwhelmingly herbaceous and the only shrub species encountered (*Amorpha fruticosa* L.) was primarily cultivated with only one wild individual found, the 5 m × 5 m protocol yielded only a single record.)

Opportunistic Presence Recording (Around Grids): To maximise spatial coverage beyond the systematic plots, any invasive plants observed during travel to/from or in the vicinity of the grid point, but outside the formal 300 m × 300 m boundary, were also recorded. For these opportunistic observations, we collected presence-only data, documenting the species identity and precise GPS coordinates without establishing a quantitative plot.

2. Roadside Transect Survey: This method was specifically designed to sample linear dispersal corridors and anthropogenically disturbed habitats along roads, which are key pathways for invasion.

Transect Establishment: The road sections we selected met two criteria: (a) a minimum continuous length of 5 km to ensure spatial representativeness and (b) an absence of major recent disturbances, such as ongoing construction. To avoid bias, one side (left or right) of each selected road section was randomly chosen for survey.

Systematic Point Sampling: Along the surveyed side of the road, a sampling point was marked at 1 km intervals. A two-phase protocol was followed at each point: Quantitative Sampling: A visual survey was conducted within the "road-effect zone" of the sampling point (a band extending approximately 20-50 m from the road edge). At locations where invasive plants were found to be concentrated, 3 - 5 quadrats were established. All invasive species within each quadrat were recorded and their percentage cover was estimated.

Opportunistic Presence Recording: For invasive plants encountered along the road, but not within the immediate area of the sampling points, the species name and GPS coordinates were recorded.

## Geographic coverage

### Description

Xiaoxing'an Mountains, China

**Figure 1.**
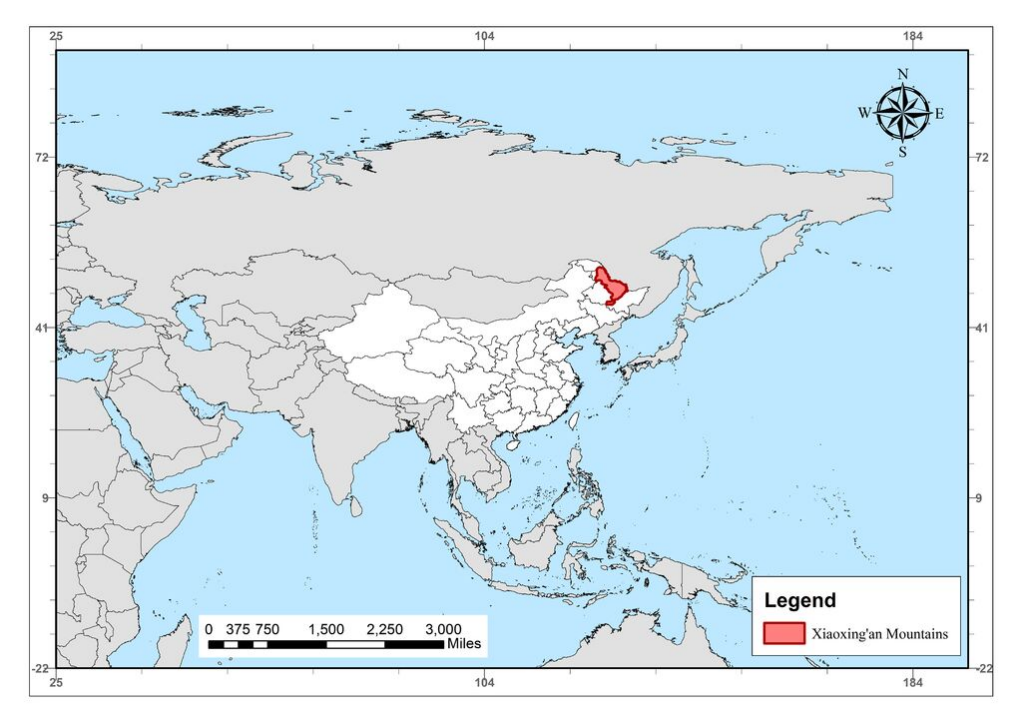
Geographic location of the Xiaoxing'an Mountains.

Fig. 1 provides the geographical context for the study area, showing the Xiaoxing'an Mountains (highlighted in red) located in northeast China, bordering the Russian Far East, within the broader context of East/Northeast Asia. This region serves as a crucial boundary zone for the study of invasive species expansion and climate warming effects.

**Figure 2.**
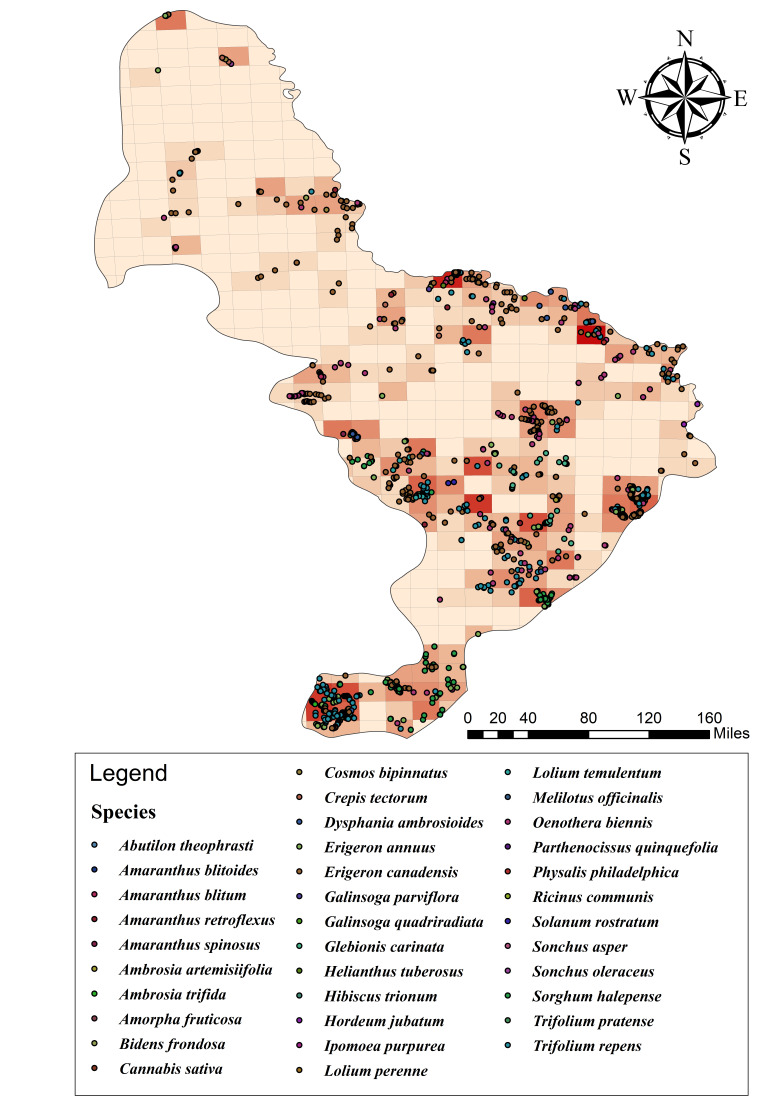
Spatial distribution of invasive plant occurrence records in the Xiaoxing'an Mountains.

The spatial pattern of invasive plant occurrences across the Xiaoxing'an Mountains is visually represented by the distribution map (Fig. 2). The majority of occurrence records and areas with the highest potential species richness are concentrated in the southern and eastern lowland areas of the mountains. Specifically, the distribution pattern is strongly aggregated along major river valleys and near urban and semi-urban settlements, demonstrating a clear correlation between invasion fronts and high anthropogenic disturbance levels, consistent with typical invasion ecology theories.

### Coordinates

45.19753 and 52.05595 Latitude; 125.61113 and 130.78148 Longitude.

## Taxonomic coverage

### Description

This study documented distribution records for 38 invasive plant species across the entire Xiaoxing'an Mountains region. The recorded alien flora encompassed 11 families, with Asteraceae accounting for the highest proportion (36.8%), followed by Amaranthaceae (13.2%), Poaceae (13.2%) and Fabaceae (13.2%). Solanaceae (5.3%) and Malvaceae (5.3%) represented relatively lower proportions. Five families were represented by only a single species and two families (Euphorbiaceae, Vitaceae) had only one observation record each (Table 1). The taxonomic classification of species in this study follows the Catalogue of Life China: Towards an index of known species present in China (Lin et al. 2025).

The species with the highest number of records was *Erigeron canadensis* (1723 records), followed by *Trifolium repens* (1157 records). *Oenothera biennis*, *Sorghum halepense* and *Erigeron annuus* were less numerous, each with approximately 500 records. This was followed by *Dysphania ambrosioides* (117 records). The remaining 32 plant species each had no more than 100 records.

## Temporal coverage

### Notes

2022–2023

## Usage licence

### Usage licence

Creative Commons Public Domain Waiver (CC-Zero)

## Data resources

### Data package title

Distribution and Abundance Dataset of Invasive Plants in the Xiaoxing'an Mountains, Heilongjiang (2022–2023)

### Resource link

https://www.gbif.org/dataset/41c1c15f-fff8-45a8-a781-b1b24d81b9cc

### Alternative identifiers

https://ipt.taibif.tw/resource?r=lesser_khingan_invasives

### Number of data sets

1

### Data set 1.

#### Data set name

Distribution and Abundance Dataset of Invasive Plants in the Xiaoxing'an Mountains, Heilongjiang (2022–2023)

#### Download URL

https://ipt.taibif.tw/archive.do?r=lesser_khingan_invasives&v=1.5

**Data set 1.** 

Column label	Column description
id	[DwC Structure: Event Core, Occurrence Extension] Internal unique identifier for the data file. In the Event file, the value is identical to eventID. In the Occurrence Extension file, this field also contains the value of eventID, serving as the link back to the core record.
eventID	[DwC Structure: Event Core, Occurrence Extension] Linking Core Identifier. A globally unique identifier for each sampling event. Used to link species occurrence records (Occurrence Extension) back to the sampling metadata (Event Core).
parentEventID	[DwC Structure: Event Core] Describes the larger, parent-level sampling activity or area that the sampling event (eventID) belongs to. In this dataset, it is used to refer to the unique ID of the 20 km × 20 km grid cell.
eventDate	[DwC Structure: Event Core] The exact date the sampling event occurred, formatted as YYYY-MM-DD.
samplingProtocol	[DwC Structure: Event Core] Describes the methodology used to collect the data.
sampleSizeValue	[DwC Structure: Event Core] The magnitude of the sampling effort.
sampleSizeUnit	[DwC Structure: Event Core] The unit of the sampling effort magnitude.
countryCode	[DwC Structure: Event Core] The code of the country where sampling occurred, consistently CN.
country	[DwC Structure: Event Core] The full name of the country where sampling occurred, consistently China.
decimalLatitude	[DwC Structure: Event Core] The latitude coordinate of the sampling event's centre point, expressed in decimal degrees.
decimalLongitude	[DwC Structure: Event Core] The longitude coordinate of the sampling event's centre point, expressed in decimal degrees.
geodeticDatum	[DwC Structure: Event Core] The geodetic system used for geographical coordinates, uniformly WGS84.
coordinateUncertaintyInMeters	[DwC Structure: Event Core] The radius of the precision of the coordinates, meaning the area the actual location might fall into, uniformly 10 meters.
occurrenceID	[DwC Structure: Occurrence Extension] A globally unique identifier for the species occurrence record. Used to uniquely identify each row in the Occurrence extension file.
basisOfRecord	[DwC Structure: Occurrence Extension] Describes the type of record, uniformly Human Observation.
organismQuantity	[DwC Structure: Occurrence Extension] The quantitative value of species abundance. In this dataset, this field records the value of Percentage Cover.
organismQuantityType	[DwC Structure: Occurrence Extension] Describes the unit of the abundance value, uniformly percentage cover.
scientificName	[DwC Structure: Occurrence Extension] The standard scientific name (Latin name) of the species.
kingdom	[DwC Structure: Occurrence Extension] The taxonomic kingdom level, uniformly Plantae.
phylum	[DwC Structure: Occurrence Extension] The taxonomic phylum level.
class	[DwC Structure: Occurrence Extension] The taxonomic class level.
order	[DwC Structure: Occurrence Extension] The taxonomic order level.
family	[DwC Structure: Occurrence Extension] The taxonomic family level.
genus	[DwC Structure: Occurrence Extension] The taxonomic genus level.
taxonRank	[DwC Structure: Occurrence Extension] The lowest taxonomic rank recorded for the species.

## Figures and Tables

**Table 1.** Number of alien plant species per family and number of occurrence records included in the distribution database.

**Plant Family**	**Number of alien species**	**Number of occurrence records**
Asteraceae	14	2406
Fabaceae	5	1177
Poaceae	5	675
Onagraceae	1	593
Amaranthaceae	5	247
Solanaceae	2	32
Malvaceae	2	15
Convolvulaceae	1	4
Cannabaceae	1	2
Euphorbiaceae	1	1
Vitaceae	1	1
